# The complete chloroplast genome of *Lonicera acuminata* Wall. and its phylogenetic analysis

**DOI:** 10.1080/23802359.2022.2073836

**Published:** 2022-05-10

**Authors:** Chenju Yang, Chunyan Jiang, Shaoxiong Wu, Xiayu Feng, Zhengwen Yu

**Affiliations:** School of Life Sciences, Guizhou Normal University, Guiyang, China

**Keywords:** *Lonicera acuminata*, Caprifoliaceae, complete chloroplast genome, phylogenetic

## Abstract

*Lonicera acuminata* Wall. is a medicinal and edible homologous plant in folk medicine that displays excellent pharmacological activities. However, the phylogenetic relationship between *L. acuminata* and other related family members remains unclear. In this study, we assembled the chloroplast genome of *L. acuminata*. The circular chloroplast genome was 154,282 bp in size, including a large single-copy region of 88,373 bp and a small single-copy region of 18,455 bp, which were separated by two inverted repeat regions (23,727 bp each). A total of 128 genes were predicted, including 8 ribosomal RNAs, 37 transfer RNAs and 83 protein-coding genes. The phylogenetic analysis revealed that *L. acuminata* was clustered together with *L. pampaninii*, *L. macranthoides* and *L. hypoglauca*.

*Lonicera acuminata* Wall. 1824 belongs to the family Caprifoliaceae, which is mainly distributed in Sichuan, Guizhou, Yunnan, Taiwan and other places in China (Gou and Wan 2005). In addition, *L. acuminata* is mainly used for carbuncles and treating sores, erysipelas, wind-heat, cold and fever because of its clearing heat and detoxifying, antibacterial and antiviral effects (Wei et al. 2010; Yang et al. 2010; Zheng et al. 2012; Liu et al. 2020; Qiu et al. 2020). In the new edition of Flora of China, *L. acuminata* and *L. pampaninii* have been merged into a single species because there is little difference in plant morphology (Chinese Academy of Science Flora of China Editorial Board 2013). However, due to the lack of molecular evidence, this study intends to provide a reference for the taxonomic merging of the two species at the molecular level.

Young and healthy leaf samples were collected by Chenju Yang from Hekou Village, Muxi Town, Muchuan County, Sichuan Province, China (28°51′7.40″N, 103°50′55.46″E, 1114 m above sea level). No specific permissions were required for the collection of plant material. The plant was identified by Dr. Chunyan Han, Kunming Caizhi Biotechnology Co. Ltd, Kunming, Yunnan, China. A specimen was deposited at a local herbarium of School of Life Sciences, Guizhou Normal University (Chenju Yang; e-mail yangchenju1123@163.com) under voucher number GZNUYCJ202105001. Total genomic DNA (No. YX20210511901) was extracted using an E.Z.N.A^®^ plant DNA kit (FEIYANG, Guangzhou, China) and stored at −80 °C in the laboratory (room number: 1403) of the School of Life Sciences, Guizhou Normal University. A total amount of 1000 ng DNA per sample was used as input material for the DNA sample preparations. The DNA library was constructed using the TruseqTM RNA Sample Prep Kit. Total DNA was used to generate libraries with an average insert size of 400 bp. The library preparations were sequenced on an Illumina platform, and approximately 3 GB of 150-bp paired-end reads were obtained and saved in fastq format. The quality of the raw data obtained was checked using FastQC for single base quality, base content distribution, GC content distribution, and sequence base quality. After the removal of adapter sequences, the filtered reads were assembled using the program GetOrganelle (Jin et al. 2020) with *Lonicera japonica* (GenBank accession number: MH028738) as the initial reference genome, and the assembled chloroplast genome was annotated using the online software GeSeq (Tillich et al. 2017). Finally, the accurate, annotated, complete chloroplast genome was submitted to GenBank with accession number MZ901373.

The length of the complete chloroplast genome sequence of *L. acuminata* was 154,282 bp, which comprised a large single-copy (LSC, 88,373 bp) region, a small single-copy (SSC, 18,455 bp) region and two inverted repeat (IRA and IRB) regions of 23,727 bp. In total, 128 genes were predicted, including 83 protein-coding genes (PCGs), 8 rRNA genes and 37 tRNA genes. Among these assembled genes, 4 rRNAs (*rrn4.5*, *rrn5*, *rrn16* and *rrn23*), 4 PCGs (*rps7*, *rps12*, *ndhB* and *ycf2*) and 7 tRNAs (*trnA-UGC*, *trnG-GCC*, *trnI-GAU*, *trnL-CAA*, *trnN-GUU*, *trnR-ACG* and *trnH-GUG*) were double copies. One tRNA (*trnV-GAC*) occurred in three copies. Intron–exon analysis showed the majority (107 genes, 84%) of genes didn't contain introns, whereas 21 (16%) genes contained introns.

To further understand the phylogenetic position of *L. acuminata*, 21 chloroplast genome sequences of the family Caprifoliaceae and one outgroup (*Sinadoxa corydalifolia*) were downloaded from GenBank to construct phylogenetic trees with *L. acuminata* through maximum-likelihood (ML) method. The ML tree based on the GTR + G + I model was constructed using IQ-tree 2.04 with 1000 bootstrap replicates (Nguyen et al. 2015). The phylogenetic analysis revealed that *L. acuminata* was clustered together with *L. pampaninii*, *L. macranthoides* and *L. hypoglauca* (Figure 1).

**Figure 1.**
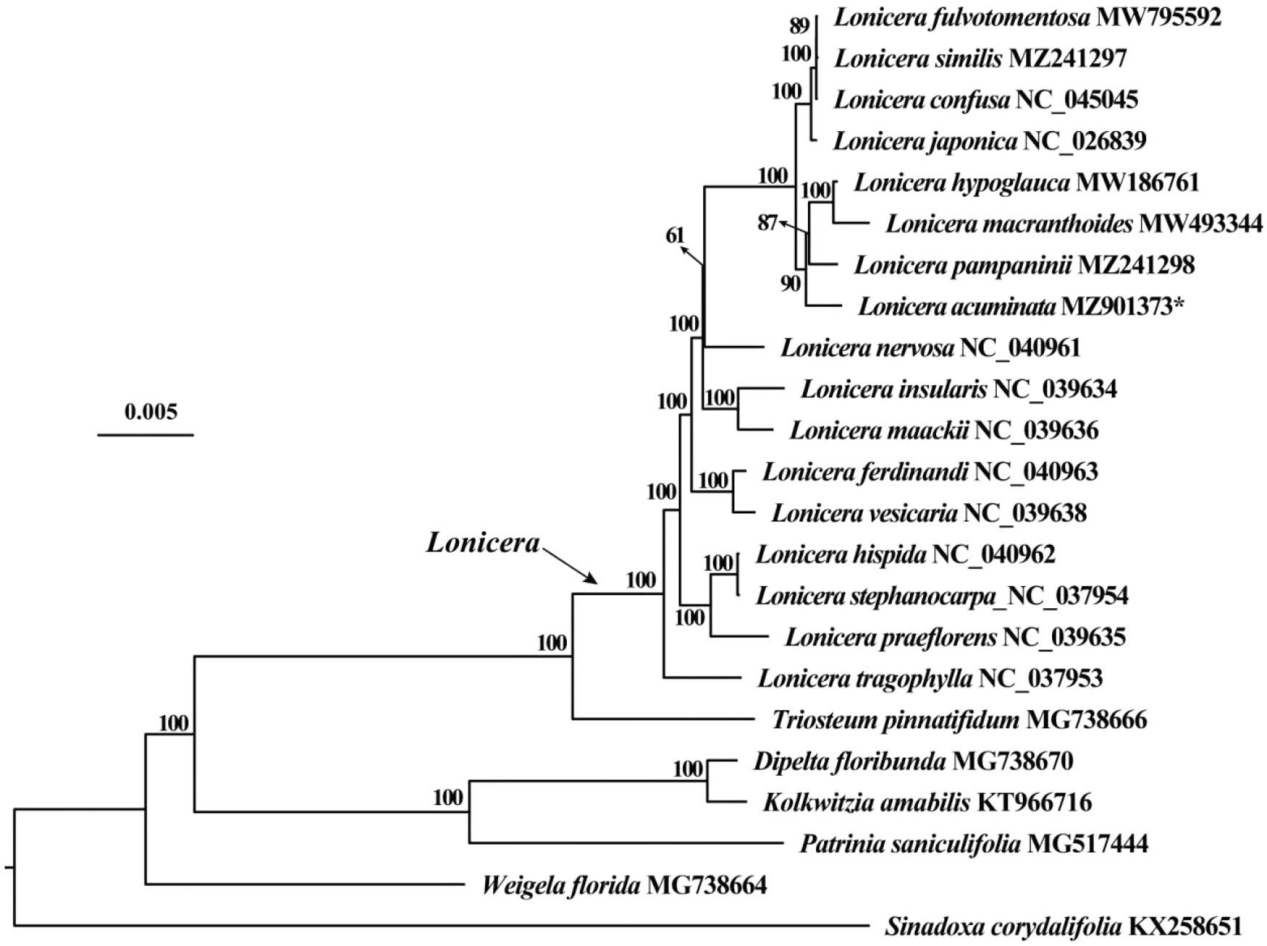
Maximum-likelihood tree based on the complete chloroplast genome sequences of 23 species. GenBank accession numbers are described in the figure. Shown next to the nodes are bootstrap support values based on 1000 replicates.

In the new edition of Flora of China, the scientific name of *L. pampaninii* has been changed to *L. acuminata*, but in our phylogenetic tree they are treated as two separate clades, so here we suggest that *L. pampaninii* and *L. acuminata* should still be treated as two independent valid species.

## Data Availability

The genome sequence data that support the findings of this study are openly available in GenBank of NCBI at https://www.ncbi.nlm.nih.gov under the accession number MZ901373. The associated BioProject, SRA, and Bio-Sample numbers are PRJNA756814, SRR15561335, and SAMN20926716 respectively.
